# Bitot-like Spots and Congenital Aniridia: A Case Report

**DOI:** 10.3390/jcm14030987

**Published:** 2025-02-04

**Authors:** Valeria Mocanu, Raluca Horhat, Florin-Raul Horhat, Mihai Poenaru-Sava

**Affiliations:** 1 Department of Ophthalmology, “Victor Babes” University of Medicine and Pharmacy, 300041 Timisoara, Romania; mocanu.valeria@umft.ro (V.M.); mihai.poenaru-sava@umft.ro (M.P.-S.); 2 Clinic of Pediatric Surgery, Emergency Children’s Hospital Louis Turcanu, 300011 Timisoara, Romania; 3 Ophthalmo-ENT Tumor Sensory Research Center (EYE ENT), “Victor Babes” University of Medicine and Pharmacy, 300041 Timisoara, Romania; 4 Department of Functional Sciences, “Victor Babes” University of Medicine and Pharmacy, 300041 Timisoara, Romania; 5 Center for Modeling Biological Systems and Data Analysis, “Victor Babes” University of Medicine and Pharmacy, 300041 Timisoara, Romania; 6 Department of Mathematics, Polytechnic University of Timisoara, 300006 Timisoara, Romania; rhorhat@umft.ro

**Keywords:** congenital aniridia, glaucoma, benzalkonium chloride, dry-eye syndrome

## Abstract

**Background:** Bitot’s spots, defined as white foamy triangular or round-shaped spots with the base located at the temporal limbus and the apex towards the lateral canthus, were initially associated with vitamin A deficiency (VAD). More recently, Bitot’s spots were also described in patients with normal vitamin A levels, associated with aniridia, dry-eye syndrome and post-thermal or chemical injury, as well as the usage of benzalkonium chloride (BAK) eyedrops. The aim of this article is to present the management of Bitot-like spots in a patient with congenital aniridia. **Methods:** An 8-year-old female patient with type 1 congenital aniridia, glaucoma, cataract, strabismus, congenital nistagmus and aniridia-associated keratopathy presented with changes in conjunctival appearance. The ophthalmological examination revealed Bitot-like spots with a foamy appearance, triangular shape, temporal location and proximity to the limbus. Further investigations were required in order to identify the cause of Bitot-like spots. Vitamin D deficiency, dry-eye syndrome, birch and Phleum genus pollen allergy were diagnosed. The patient underwent oral medication with vitamin D and topical treatment with steroids eye solution, preservative-free artificial tears and vitamin A ointment. **Results:** After three months of treatment, we observed the disappearance of the Bitot-like spots. **Conclusions**: Congenital aniridia, but also its complications such as glaucoma, dry-eye syndrome and the use of benzalkonium chloride topical medication, increases the risk of Bitot-like spots.

## 1. Introduction

According to the National Organization for Rare Disorders (Orphanet), congenital aniridia is a rare disease [1], with an estimated incidence of 1:40,000 to 1:100,000 [2]. Approximately two-thirds of the cases are familial forms, with an autosomal dominant inheritance pattern of Pax-6 gene abnormalities and more rarely with an autosomal recessive one, in the context of the Gillespie syndrome [3,4,5].

Aniridia represents the congenital absence of the iris. It is, however, a pan-ocular disease, involving the cornea, iris, anterior chamber angle, lens and fovea. It is usually associated with nystagmus, cataract, glaucoma and foveal hypoplasia [3].

A vast majority of the patients exhibiting Pax-6 mutations develop aniridia-associated keratopathy (AAK), with impaired vision and painful progressive conjunctivalization of the corneal surface. Previous studies have found an association between the severity of the corneal changes and the reduction in the tear production [6] or the instability of the tear film [3,7].

Bitot’s spots are white foamy triangular or round-shaped spots, with the base located at the temporal limbus and the apex towards the lateral canthus. They were first described by Pierre Bitot in 1863, in association with xerophthalmia and vitamin A deficiency (VAD). As a part of Bitot’s spots, the deficiencies lead also to ocular manifestations such as night blindness, conjunctival and corneal xerosis, keratomalacia and corneal ulceration [8]. Vitamin A deficiency may be caused by reduced intake (malnutrition, dysphagia, alcoholism or mental illness), absorption disorders (Crohn’s disease, celiac sprue, pancreatic insufficiency or short bowel syndrome), transport deficiency (abetalipoprotenemia) or reduced storage (liver disease) [8,9].

Protein malnutrition induces reduced retinol-binding protein (RBP) important for the release of vitamin A by the liver, blood transportation and tissue absorption [8]. In the absence of VAD, protein malnutrition is unlikely to cause xerophthalmia. Diarrhea, respiratory tract infection with fever, measles and chicken pox induce the deficiency of serum vitamin A by decreased absorption and increased excretion. Worm infestation may be associated with VAD because of decreased absorption [8].

More recently, Bitot’s spots were also described in patients with normal vitamin A levels and anterior segment pathologies including aniridia, exposure to hot climates (drying ocular surface), vitamin B deficiency, post-thermal or chemical injury and post-use of benzalkonium chloride (BAK) eyedrops, as well as in otherwise normal ocular examination [3].

Overall, there are only four cases mentioned in the literature of Bitot’s spots in aniridia patients in the absence of VAD. All of them were also associated with glaucoma, and in three of them, preservative ocular hypotensive drugs were administered [10].

## 2. Detailed Case Description

### 2.1. Patient’s Presentation

In June 2024, an 8-year-old female patient presented to the Emergency Children Hospital Timisoara, Romania, with bilateral changes in the conjunctival appearance.

The patient was diagnosed at birth with congenital aniridia type 1 confirmed by the c.1309dup mutation in the Pax-6 gene, strabismus and nistagmus. She was also diagnosed with intellectual disability, but genito-urinary abnormalities were absent.

The patient underwent bilateral glaucoma surgery when she was age 4 and since then she followed a treatment with carbonic anhydrase inhibitor/timolol and prostaglandin analogue eyedrops for glaucoma.

One year after the surgery, the patient was diagnosed with dry-eye syndrome, for which preservative-free artificial tears with hyaluronic acid were prescribed. The first corneal alterations were reported in 2023, marking the onset of the AAK, along with lens opacities.

Written informed consent has been obtained from the patient’s caregivers in order to perform the necessary investigations, as well as to publish this paper.

### 2.2. Ophthalmological Examination

**Figure 1:**
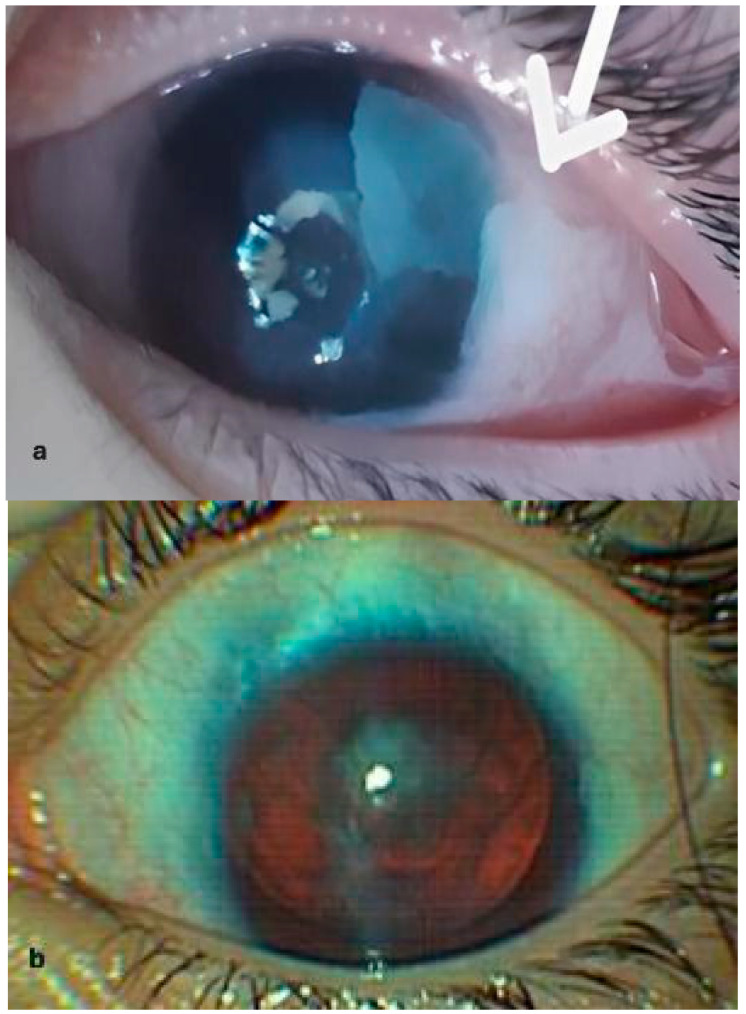
(**a**) Foamy, white, triangular Bitot’s spot on the temporal conjunctiva at diagnosis. (**b**) Aniridia, cataract and aniridia associated keratopathy (AAK) grade 2, with onset of central corneal area opacification and vessels converging towards opacity in the left eye, at 6 months after the initial diagnosis.

A complete ophthalmological examination was undertaken. The slit lamp examination has shown indistinguishable clinical Bitot’s spots with a foamy appearance, triangular shape, temporal location and proximity to the limbus and stage 2 AAK in both eyes, with conjunctivalization of the peripheral and paracentral cornea, vascular pannus and a clear central cornea (Figure 1). The fundus examination revealed foveal hypoplasia and partial optic atrophy. Decimal visual acuity was 0.1, unchanged from the 2 months prior examination. The intraocular pressure was within normal range, under specific treatment for glaucoma.

Taking into account the patient’s history, the tear film was reevaluated.

In order to diagnose dry-eye syndrome, we used the criteria described by Jastaneiah [7]. For the positive diagnosis, at least 4 out of the 6 proposed criteria must have abnormal values: (1) Schirmer’s test without anesthesia (>25 mm, normal) and with anesthesia (>15 mm, normal), (2) TBUT (>10 s, normal), (3) tear meniscus level (>0.5 mm, normal), (4) conjunctival and corneal fluorescein staining, (5) corneal rose Bengal and (6) presence of mucoid secretions [7]. The fluorescein conjunctival and corneal staining was evaluated according to the Oxford grading scale [11].

The Schirmer test I revealed values of 6 mm for the right eye (RE) and 7 mm for the left eye (LE), suggestive of dry-eye syndrome. The test was performed using 0.4% oxybuprocaine hydrochloride (Rompharm Company, Bucharest, Romania) as a topical anesthesia and inserting the Schirmer strips (Madhu Instruments Pvt., Ldt., New Delhi, India) into the lower conjunctival sac without touching the cornea. 

To evaluate the tear breakup time (TBUT), we instilled fluorescein into the patient’s tear film. The patient was asked not to blink in order to observe the tear film at the slit-lamp with cobalt blue illumination. We measured the seconds elapsed from the last blink to the appearance of the first dry spot. The result of the TBUT was 5 s for the RE and 4 s for the LE.

For the tear meniscus level, a horizontal slit beam was used to match the tear meniscus height centrally along the lower lid. The tear meniscus level was 0.3 mm in both eyes.

For the fluorescein staining, a fluorescein strip was used (Madhu Instruments Pvt., Ltd., New Delphi, India) with topical anesthetic (0.4% oxybuprocaine hydrochloride, Rompharm Company, Bucharest, Romania) in order to identify under cobalt blue light, abnormal and missing epithelial cells in the cornea and temporal and nasal conjunctiva. According to the Oxford grading scale, grade II suggests that mild severity alterations were observed.

It was also noted the presence of mucoid secretions.

We were unable to perform rose Bengal testing because of the lack of product.

### 2.3. Laboratory Tests and Supplementary Investigations

The main causes of Bitot’s spot appearances were systematically investigated.

From the patient’s medical history, no infection, fever or diarrhea was diagnosed in the last 6 months.

Taking into account the Pax-6 gene mutation, in order to exclude Wilms tumour, a pediatric consult was required. A gastroenterological consult was also performed in order to exclude a possible malabsorption syndrome.

The complete blood count, C reactive proteins and erythrocyte sedimentation rate values within the normal range excluded infection. Liver and kidney functions were unaffected, as shown by the normal aminotransferases (ALAT, ASAT) and creatinine levels.

Vitamin A deficiency was ruled out, as the serum vitamin A, as well as the serum retinol-binding protein (RBP) were within the normal range.

In order to identify other vitamin or microelement deficiencies, additional tests were performed (see Table 1). The latter revealed vitamin D deficiency and a Birch and Phleum genus pollen allergy.

### 2.4. Treatment and Evolution

Oral vitamin D supplements, steroids eyedrops, preservative-free artificial tears (containing cross-linked hyaluronic acid, trehalose and stearylamine in liposomes) and vitamin A ointment were added to the glaucoma treatment (Table 2). Exposure to electronic devices was limited to 1 h per day. Monthly follow-ups were undertaken.

The disappearance of Bitot-like spots was observed after three months of treatment. At the three-month follow-up, the Schirmer test I with topical anesthesia showed 12 mm for the RE and 11 mm for LE. The result of the TBUT was 10 s for the RE and 9 s for the LE and the tear meniscus level was 0.5 mm in both eyes. The absence of fluorescein staining and mucoid secretions were noted.

## 3. Discussion

Bitot’s spots are represented by conjunctival metaplasia into keratinized squamous epithelium, classically associated with vitamin A deficiency, although they were found to also be caused by other vitamin deficiencies, such as vitamin B [12,13], or to appear in patients living in hot climates, with chronic ultraviolet or dust exposure [14,15,16].

The presence of Bitot’s spot with normal levels of vitamin A associated with anterior segment pathology was mentioned for the first time by Maudgil et al., who analyzed 10 retrospective cases. The Pax-6 mutation was present in 25% of the cases with unilateral lesions and 67% of those with bilateral localization. Aniridia was reported in 40% of the patients (Table 3). The same study included six patients with glaucoma treated with a combination of carbonic anhydrase inhibitor/timolol and prostaglandin analogue eyedrops [10].

Although the reported patient had normal vitamin A levels, she was diagnosed with Pax-6 mutation-positive aniridia and was under treatment with hypotensive eyedrops. It was previously suggested that BAK preservatives can cause tear film instability and squamous metaplasia. The use of benzalkonium chloride in multidose topical ophthalmic medications is essential in order to maintain sterility. Studies reported cytotoxic damage to conjunctival and corneal epithelial cells, such as the loss of conjunctival goblet cells, lymphocyte infiltration of conjunctival epithelium, increased levels of inflammatory markers as well as reduced survival of corneal and conjunctival cells [17]. Due to the presence of BAK in ocular topical treatment, the patients may present clinical manifestations of ocular surface disease: ocular dryness, foreign body, burning and/or itching sensation. These clinical manifestations have been reported in 30–70% of glaucoma patients with chronic BAK exposure [18].

When using Schirmer’s test, abnormal values were suggestive of dry-eye syndrome. This might be associated with the use of carbonic anhydrase inhibitor/timolol and prostaglandin analogue eyedrops, but also with the underlying pathology. Previous research has found an association between aniridia and dry-eye syndrome.

Dry-eye syndrome is a complex tear film disorder, determined by quantitative and qualitative changes. It consists of three different layers, each with a specific function. The mucin inner layer is generated by the goblet and the corneal epithelial cells and acts as a barrier against microtrauma. The lacrimal glands synthetize the middle aqueous layer and their function is evaluated by the Schirmmer test. The Meibomian glands produce the lipid layer, which delays evaporation.

A correct evaluation of dry-eye syndrome associated with anirida should aim to investigate all three components of the tear film.

Mackman et al. observed decreased Schirmer test results in five of eight patients [6]. Jastaneiah et al. reported dry eyes in 34 of 36 aniridia eyes. Tear breakup time was less than 5 s in 80.6% of the cases and tear meniscus was reduced to less than 0.5 mm in 88.6% of the patients. The same study concluded that the majority of aniridia patients suffer from moderate-to-severe dry-eye syndrome [7]. Perat et al. reported reduced TBUT and increased corneal staining in 15 aniridia patients [19]. Landsend et al. identified increased ocular surface staining, Meibomian gland loss and modified tear film osmolarity in 35 aniridia patients [11]. Shiple et al. reported dry-eye syndrome in 55 of 99 aniridia patients (56%) concluding that the ocular surface problem in congenital aniridia is a multifactorial one [20].

Dry-eye syndrome associated with aniridia or to allergy can be a possible etiology for Bitot’s spot in our patient. Therefore, the ongoing treatment with carbonic anhydrase inhibitor/timolol and prostaglandin analogue eyedrops was supplemented with steroid and artificial tears containing trehalose and stearylamine in liposomes. After three months of treatment, Schirmer’s test showed improved symptoms, and the Bitot-like spots disappeared.

The case we reported is the fifth in the literature featuring aniridia and Bitot-like spots, with normal vitamin A levels. An additional case with the same association was described by Shetti et al.; however, the patient was diagnosed with VAD [2]. The uniqueness of our case consists of the fact that, in addition to the anterior segment pathology and the usage of preservative eyedrops, we also identified in our patient the presence of dry-eye syndrome and Birch and Phleum genus allergy.

When Bitot’s spots appear in VAD, the lack of vitamin A administration can lead to severe complications such as corneal xerosis, keratomalacia, corneal scar, xerophthalmia and significant visual loss [21].

In our case, no VAD was identified. A multifactorial etiology was diagnosed: the presence of dry-eye syndrome, aniridia, allergy and use of BAK ocular hypotensive medication.

## 4. Conclusions

We reported a rare case of Bitot’s spot, with normal vitamin A levels, associated with aniridia, glaucoma, dry-eye syndrome and use of BAK-topical medication.

A detailed diagnosis of dry-eye syndrome and the targeted treatment of the tear film dysfunction, as well as the use of preservative-free eye-drops led to the disappearance of the lesions in our patient.

## Figures and Tables

**Table 1:** Laboratory tests for vitamin and microelements deficiencies, allergies and possible causes of malabsorption.

Laboratory Test	Patient Result	Normal Values
Serum vitamin A	0.31 mg/L	0.26–0.49 mg/L
Serum RBP	40 mg/L	30–60 mg/L
Serum zinc	0.81 mg/L	0.65–2.56 mg/L
Folic acid	29.81 nmol/L	>12.19 nmol/L
Serum vitamin B12	314 pmol/L	156–672 pmol/L
25-hydroxy vitamin D *	21.9 ng/mL	30–100 ng/mL
Immunoglobulin E (Ig E)	21.34 Ul/mL	0–90 Ul/mL
Allergen-specific immunoglobulin E *	Very low concentration (0.35–0.7 kU/L) for Birch pollen and Phleum genus pollen	Normal < 0.35 kU/L
Immunoglobulin A (Ig A)	0.84 g/L	0.34–3.05 g/L
Immunoglobulin G (Ig G)	11.43 g/L	5.72–14.74 g/L
Anti-transglutaminase antibodies (tTG)		
tTG-IgA	1.07 U/mL	Negative < 4 U/mL
tTG-IgG	1.42 U/mL	Negative < 20 U/mL
IgA anti-endomysial antibodies	negative (<1:5)	negative (<1:5)
IgG anti-endomysial antibodies	negative (<1:5)	negative (<1:5)
Stool digestive test	Positive for starch, fat	
Stool occult bleeding test	negative	negative
Helicobacter Pylori Antigen stool test	negative	negative
Coproparasitological examination (micro-/macro-scopic examination)	absence of parasitic elements	absence of parasitic elements

* Blood test with values outside the normal range.

**Table 2:** Patient’s treatment before and after Bitot’s spots diagnosis.

	Before Diagnosis	After Diagnosis
Ocular hypotensive medication		
1. carbonic anhydrase inhibitor/timolol	Benzalkonium Chloride + Dorzolamide + Timolol	Dorzolamide + Timolol preservative free 2 × 1 drops/day
2. prostaglangin analogue	Latanoprost preservative-free	Latanoprost preservative-free (1 drop/day)
Artificial tears	Hyaluronic acid 0.15% preservative-free	Cross-linked hyaluronic acid 0.15%+ trehalose3%+ stearylamine in liposomes 0.25% (4 × 1 drops/day)
Steroid eye drops	-	Fluorometholone 2 mg/mL (4 × 1 drops/day for 7 days, followed by 3 × 1 drops/day for 14 days, then 2 × 1 drops/day for 14 days and finally 1 drop/day for 7 days)
Vitamin A	-	Vitamin A 250 IU/g preservative-free eye ointment, 1 topical applic./day, in the evening
Vitamin D	-	Colecalciferol 500 UI/day

**Table 3:** Cases of Bitot-like spots and anirida with normal vitamin A levels in the literature.

Author	Age	Genetic Anomaly	Glaucoma	Preservative Eyedrops-Drops/Day, Duration	Laterality
Maudgil et al. [10]	7	PAX6 mutation c.379_380delCTinsG	Yes	8–10, 4 yrs.	bilateral
Maudgil et al. [10]	10	PAX6; 11p14.1p12 deletion	Yes	5, 5 yrs.	bilateral
Maudgil et al. [10]	7	PAX6; 11p14.1p13 deletion (WAGR syndrome)	Yes	-	bilateral
Maudgil et al. [10]	11	PAX6; 11p13p11.2 deletion (WAGR syndrome)	Yes	5, 7 yrs.	unilateral
Mocanu et al. (present case)	8	PAX6; c.1309dup	Yes	3, 4 yrs.	bilateral

## Data Availability

Additional patient data can be obtained from the authors upon reasonable request. The data are not publicly available due to privacy concerns.
